# Dynamic Service Function Chaining Orchestration in a Multi-Domain: A Heuristic Approach Based on SRv6

**DOI:** 10.3390/s21196563

**Published:** 2021-09-30

**Authors:** Yutong Wu, Jinhe Zhou

**Affiliations:** 1 Key Laboratory of the Ministry of Education for Optoelectronic Measurement Technology and Instrument, Beijing Information Science and Technology University, Beijing 100192, China; yutongwu97@bistu.edu.cn; 2 School of Information and Communication Engineering, Beijing Information Science and Technology University, Beijing 100101, China

**Keywords:** Network Function Virtualization, Service Function Chaining, SRv6, dynamic deployment, end-to-end delay, bandwidth resource consumption, load balancing

## Abstract

With the emergence of virtualization technology, Network Function Virtualization (NFV) and Software Defined Networking (SDN) make the network function abstract from the hardware and allow it to be run on virtual machines. These technologies can help to provide more efficient services to users by Service Function Chaining (SFC). The sequence of multiple VNFs required by network operators to perform traffic steering is called SFC. Mapping and deploying SFC on the physical network can enable users to obtain customized services in time. At present, a key problem in deploying SFC is how to reduce network resource consumption and load pressure while ensuring the corresponding services for users. In this paper, we first introduce an NFV architecture for SFC deployment, and illustrate the SFC orchestration process which is based on SRv6 in multi-domain scenario. Then, we propose an effective SFC dynamic orchestration algorithm. First, we use Breadth-First Search algorithm to traverse network and find the shortest path for deploying VNFs. Next, we use the improved Ant Colony Optimization algorithm to generate the optimal deployment scheme. Finally, we conduct a series of experiments to verify the performance of our algorithm. Compared with other deployment algorithms, the results show that our solution effectively optimizes end-to-end delay, bandwidth resource consumption and load balancing.

## 1. Introduction

In traditional networks, network functions are provided by different hardware devices. Network service providers (NSPs) need to deploy a large number of devices when deploying new services, which are expensive and inflexible. When services change or requests increase, corresponding devices need to be added, which increases the capital expenditure (CAPEX) and operating expenses (OPEX) [1]. Therefore, NFV/SDN technology has emerged to respond the above challenges. Software Defined Networking (SDN) technology makes the separation of control and user planes come true, bringing independent scalability and service agility [2]. The NFV/SDN architecture decouples hardware and software, and Virtual Network Functions (VNFs) make the network function virtual and migratable. All these technologies are designed to provide more efficient services to users. The service provided by NSP is usually realized by a service function chain (SFC). SFC refers to the process of steering traffic through a set of functions in order to deliver an end-to-end service [3]. Although SFC can provide customized services and improve network performance, it also brings new obstacles, namely how to orchestrate and deploy SFC efficiently.

The reshaping of the 5G core network (5GC) upgrades traditional networks. Using NFV architecture, which is similar to Service-Based Architecture, to solve SFC scheduling and deployment problems is a common method currently. Therefore, the SFC orchestration scheme in this article can be well applied to the 5G environment. The 5G core network is mainly composed of different Network Functions (NFs). In the NFV framework, NFs can be regraded as VNFs to use for SFC orchestration. Therefore, the orchestration of SFC can be transformed into a problem of VNF placement. Placing the VNF on the physical network node and mapping the virtual link to the physical link represent the orchestration and deployment of SFC.

In the existing research, several problems can be identified in the orchestration and deployment of SFC. First, most researches are focused on the static deployment of SFC. However, in real network, users’ requests for services are dynamically changing, then static SFC orchestration will lead to waste of resources and delay in services. Second, the current SFC does not combine with other emerging network technologies to optimize network performance and reduce network load, such as SRv6. Third, the existing research is mostly about SFC orchestration in a single domain, but now there are more and more services that need to be orchestrated across domains.

To overcome these difficulties, we first introduce an NFV architecture based on the 5GC SBA, then we investigate the cross-domain dynamic SFC deployment problem. It has been confirmed that this problem is an NP-Hard problem. Next, we combine the SFC deployment with the SRv6 mechanism, and design an SFC dynamic orchestration (SFCDO) algorithm. The algorithm consists of two steps. First, we use the Breadth-First Search (BFS) algorithm to traverse network topology and find the shortest path to deploy VNFs. Then, we select an Improved Ant Colony Optimization (IACO) algorithm to get the best SFC deployment scheme and to adjust the deployment scheme dynamically.

In this paper, our research objective is to dynamically deploy SFC based on the SRv6 in multi-domain scenarios, while considering the lowest bandwidth resource consumption and minimizing the end-to-end delay, and, at the same time ensuring network load balancing and service provision. In order to achieve these aims, our work makes the following contributions:

- We describe an ETSI NFV architecture that can be used in a 5G environment. Using this architecture, we can arrange the deployment of SFC, and provide customized services to different users.
- We build a mechanism for deploying SFC based on SRv6. We can use the scalability of SRv6 to deploy SFC flexibly without adding any other hardware or network equipment. Later, we explain the specific process of SFC orchestration based on SRv6. We also propose a multi-domain orchestration scenario.
- For the orchestration and deployment of SFC, we construct a joint optimization problem of end-to-end delay, resource consumption, and network load balancing, which can be proved as an NP-Hard problem.
- To solve the joint optimization problem, we propose an algorithm to achieve the optimization goals. We first adopt BFS algorithm to find the shortest physical path.Then we use IACO algorithm to deploy VNFs to physical nodes in order, which is an improved heuristic algorithm that can be used based on dynamically changing scenarios. We first adopt the BFS algorithm to find the shortest physical path for SFC deployment. Then we use the IACO algorithm to deploy VNFs to physical nodes in order. IACO is an improved heuristic algorithm that can deploy solutions based on dynamically changing scenarios.
- Finally, we compare the performance of our approach with state-of-the-art SFC dynamic deployment algorithms through a series of simulation experiments. Simulation results show that our algorithm can reduce the average end-to-end delay by 22%, average bandwidth resources consumption by 18%, and get better load balancing. Therefore, the deployment scheme proposed in this paper is a feasible solution to SFC orchestration problem.

The rest of this paper is organized as follows. In Section 2, we summarize the related work done by the previous researchers. In Section 3, we introduce the network architecture, including the multi-domain SFC orchestration and SFC deployment’s working mechanism based on SRv6. We describe the deployment problem and set up the system model in Section 4. We propose a dynamic SFC deployment algorithm in Section 5. We introduce the simulation experiments and performance evaluation of this algorithm in Section 6. Finally, we give a conclusion of this work and mention the future work in Section 7.

## 2. Related Work

In recent years, research on SFC deployment has become a hot topic. Researchers have undertaken a lot of research on the SFC deployment scheme.

In the existing research, most studies that have focused on the static SFC orchestration and deployment did not take into account that service requests are constantly changing dynamically. Sun, G et al. [4] considered an SFC orchestration of multi-domain scenarios which chose the full mesh aggregation approach to build an abstracted network. They adopted a heuristic algorithm to deploy sub-chains in multiple domains. Huin et al. [5] proposed an integer linear programming (ILP) formulation for SFC deployment problem, and they construct a decomposition model that relies on joint routing and placement configuration to deploy SFC. Savi et al. [6] put forward another ILP model to solve the SFC orchestration problem. The authors considered the impact of the network function position on the deployment costs. Gupta et al. [7,8] considered the joint problem of VNF placement and traffic routing in order to minimize network bandwidth consumption.

Some researchers have studied SFC’s deployment in different network environments, or combined its deployment with other 5G-related technologies, which have advanced to the orchestration of SFC. Paolucci et al. [9] proposed effective service chaining enforcement along traffic engineering paths combined with the SR-MPLS technique. Sebastian Troia et al. [10] proposed a mathematical model that can dynamically provide network slices on a physical network. There are a group of SFC and network resources on each network slice and they can be dynamicly deployed. The method in this article has two steps. One is to solve the VNF placement problem, and the other is to minimize the total network power consumption when considering the RWA problem. Dab et al. [11] chose to arrange the SFC in a cloud-native environment and tested its performance in the container. Sun et al. [12] proposed a DMRT-SL algorithm to map the SRs in edge computing efficiently. They proposed a novel workflow-like service request, showing an outstanding performance in terms of time delay but without considering the energy consumption. In [13], the authors investigated the application of Reinforcement Learning for performing dynamic SFC resources allocation in NFV-SDN enabled metro-core optical networks. The RL agent decides if and when to reconfigure the SFCs, which is an effective SFC resource allocation solution. Xiaoli Zhang et al. [14] proposed a verification scheme that can verify the execution of SFC in arbitrary cloud architectures. However, the above authors did not consider combining with the SRv6 mechanism that can naturally support SFC orchestration.

Some studies optimized network performance by deploying algorithms. When designing the SFC deployment optimization algorithm, other scholars considered different optimization objectives. Kuo et al. [15] considered the path length and the virtual machine reuse factor. Then they proposed a DC-LaS algorithm to find a solution whose path length and reuse factor can be improved in the SFC deployment. However, they only considered resource consumption. Liu et al. [16] proposed a two-step algorithm G-SA for SFC deployment. They first used the greedy algorithm to deploy VNFs. Then, they computed the shortest path from the source node to the destination node by simulated annealing algorithm. However, they only considered bandwidth consumption and latency. Hu and Li [17] proposed an SFC runtime framework NFCompass and ultimately reduced the length and complexity of the processing SFC, achieving better load balancing. The authors of Ref. [18] proposed three heuristic algorithms for SFC deployment: ER, ER_CS, and ER_CS_ADJ. The ER algorithm considered the reliability requirements when deploying SFC, ER_CS optimized the network load based on the above algorithm, and the ER_CS_ADJ algorithm further considered the deployment paths’ bandwidth resource consumption in the orchestration processing. The above researchers did not consider multiple metrics when they deployed SFC dynamically.

Some researchers have focused on multi-domain SFC orchestration. In [3], Toumi, N et al. developed a multi-domain SFC orchestration architecture, which utilizes the ETSI MANO standard and the principles of SDN and hierarchical SFC. Yan, Boyuan et al. [19] proposed an SFC-Oriented Topology Aggregation (SOTA) method to enable abstraction for SFs in multi-domain optical networks, then they put forward two cross-domain service function path provisioning algorithms which can reduce the blocking probability. In [20], Joshi, K.D. et al., proposed pSMART for efficient multi-domain SFC mapping, which is a lightweight, privacy-aware service function chain orchestration in a multi-domain NFV/SDN scenario. This approach efficiently optimizes the SFC orchestration response time.

Compared with the existing literature, we comprehensively consider dynamic SFC orchestration and deployment, and combine it with SRv6 technology in a multi-domain. Then we propose an algorithm specifically for SFC orchestration and deployment that can be also used in 5G mobile networks, which can effectively reduce the delay and bandwidth resource consumption of service transmission while considering network load balancing.

## 3. Network Architecture

**Figure 1:**
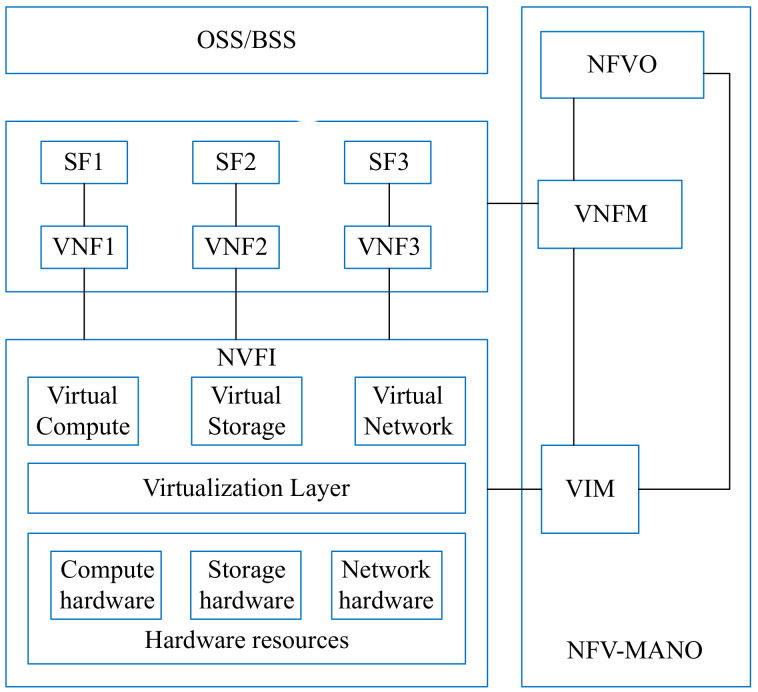
The ETSI NFV architecture.

The NFV architecture is illustrated in Figure 1. There are multiple modules in this architecture, such as NFV-MANO and NFVI [21]. Most of the components of this architecture are the same as Service-Based Architecture in 5GC.

The NFV Orchestrator (NFVO) component is responsible for managing the VNFs operations such as instantiation, deletion, and extension by using Virtualized Infrastructure Manager (VIM) [22]. The Virtual Network Function Manager (VNFM) is accountable for managing VNF instances [23]. In the traditional NFV architecture, the SDN controller uses the global view of the whole network to provide flow rules in the flow table of switches. In this architecture, we use the SDN controller to deliver the segment, which represents an ordered list of instructions [24]. According to the source routing mechanism of SR, the controller only needs to send all the information to the source node. This reduces the controller load, and other nodes do not need to maintain the state of each flow. 3GPP has introduced Network Function in 3GPP release 15 [25]. Each Network Function (NF) exposes a set of services [26]. In this paper, we assume that an NF provides a specific service. In this figure, we use VNF to represent NF, so we can say that a VNF provides a service in this deployment scheme.

In recent years, most of the research focuses on the orchestration of service function chains in a single domain, but the Internet is composed of multiple domains, which are owned and managed by different NSPs. This causes research on NFV and SFC multi-domain orchestration become essential and critical. Users also have service requirements between different domains, so cross-domain service requests are gradually increasing, and NSPs need to provide required services to users by cross-domain service function chain orchestration.

**Figure 2:**
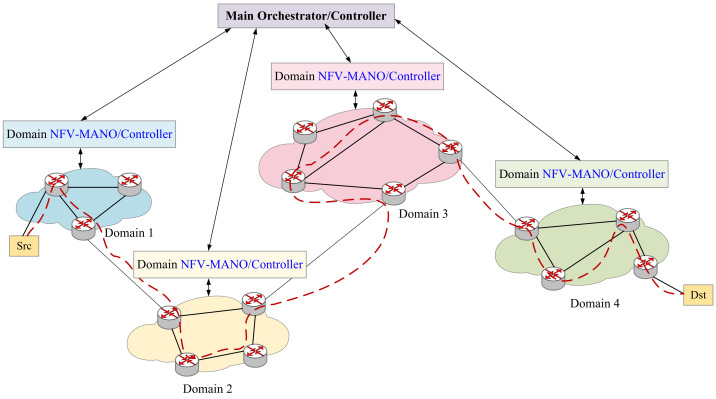
Multi-domain SFC Orchestration.

In Figure 2, we describe how an SFC is deployed across domains. First, we map each VNF in an SFC to a physical node. Secondly, we consider the source node and destination node, and then send the segment list to the source node through the SDN controller. The source node will route on the physical network routers according to these instructions, and perform the same operation when encountering a cross-domain node. When the traffic finishes through the mapping nodes in order, the deployment of an SFC is completed. Consequently, the service function chain orchestration and deployment scheme proposed in this article also can be applied to multi-domain scenarios to provide users with a wider range of orchestration schemes.

The proposed solution in Figure 2 is compliant to ETSI architecture as follows. First, the VNFM in each NFV-MANO is connected to the VNF in the virtual network and manage the VNF lifecycle; secondly, the VNF is connected to the NFVI which can provides the computing resources of the physical nodes and the bandwidth resources of the physical links. The physical node has computing/storage/network resources, which can be used for virtual node mapping. The VIM in NFV-MANO controls and manages the resources in NFVI. In addition, NFVO can directly connect to VIM for resource allocation and reservation, and collect virtualized resource configuration and status information.

**Figure 3:**
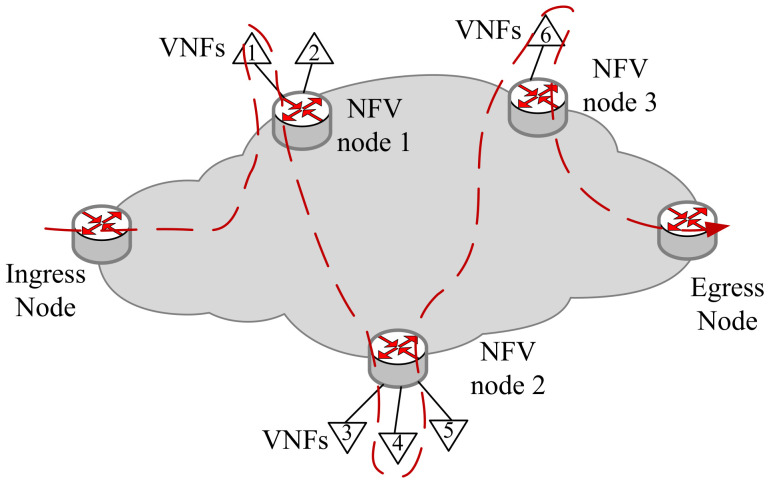
The SFC deployment based on SRv6.

Next, in Figure 3, we will illustrate the SFC orchestration based on the SRv6 mechanism in a single domain, which can be extended to multi-domain. The SRv6 architecture is a promising solution to support services like Traffic Engineering and Service Function Chaining [27]. In the SR domain, the different VNFs are hosted by NFV nodes. The packets associated with VNF chains are classified in ingress nodes, while the SR encapsulation is added [28]. The flow will pass through the VNFs on different NFV nodes in sequence. If the VNF is an SR-aware VNF, it will directly match the Segment Identifier (SID) of this VNF. Otherwise, it will match the SID of the NFV node hosting the VNF. As shown in Figure 3, after the flow leaves from the Egress node, we can get a segment list <NFV1:VNF1, NV2:VNF4, NFV3:VNF6, End>, while the SR Header (SRH) is reversed as (End, NFV3:VNF6, NFV2:VNF4, NFV1:VNF1).

**Figure 4:**
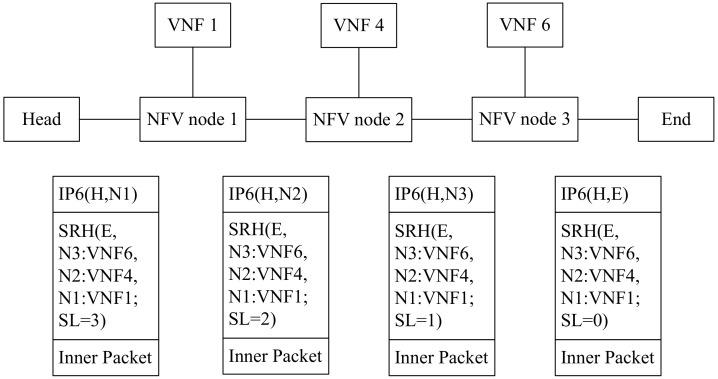
The workflow of SFC based on SRv6.

As for how data packets travel in SRv6-aware network, we describe the process in Figure 4. The unit “Head” acts as a service classifier, guarantees that these packets can travel through an SFC (VNF1 -> VNF4 -> VNF6) in order by corresponding SR policy [29]. That is why the packet is encapsulated with an IPv6 header and an SRH containing the segment list <NFV1:VNF1, NFV2:VNF4, NFV3:VNF6, En>.

When NFV node 1 receives the encapsulated packet, according to the active segment, it knows how to send the packet to the VNF. If VNF1 is SR-aware, it can process an IPv6 encapsulated packet under the guidance of SRH. So the packet is sent to VNF1 by the IP and SR headers (H, NFV2:VNF4) (E, NFV3:VNF6, NFV2:VNF4, NFV1:VNF1; SL = 2). VNF1 performs the required service function on the received packet. After the packet return from VNF1, NFV node 1 will send it to NFV node 2 according to the IPv6 destination address.

If VNF1 is SR-unaware it cannot process IPv6 encapsulated packets with an SRH, so the encapsulation headers must be stripped from the packet before it is sent to VNF1. When the packet returns from VNF1, the NFV node 1 would re-encapsulate the packet header that had been previously stripped and then send the packet to NFV node 2 according to the IPv6 destination address.

When the encapsulated packet arrives at NFV node 2, the NFV node 2 will perform a similar action to that described above.

## 4. System Model

### 4.1. Physical Network

We set the underlying physical network as an undirected graph $G=\left(V,E\right)$, where $V=\left\{v_{1},v_{2},v_{3},\cdots ,v_{\left|M\right|}\right\}$ represents the set of physical network nodes, and $E=\left\{e_{1},e_{2},e_{3},\cdots ,e_{\left|N\right|}\right\}$ denotes the set of physical network links, $\left|M\right|$ and $\left|N\right|$ represent the number of network nodes and links, respectively. $v_{i}$ represents a physical node, e.g., switch or server, which has certain computing resources $c\left(v_{i}\right)$. We use $r\left(v_{i}\right)$ to represent the remaining computing resources on physical node. $L\left(v_{i}\right)$ denotes the node load rate. We define the calculation of node load rate $L\left(v_{i}\right)$ in Formula (1).

(1) $$\begin{array}{c} L\left(v_{i}\right)=\frac{c\left(v_{i}\right)-r\left(v_{i}\right)}{c\left(v_{i}\right)},\forall v_{i}\in V \end{array}$$

For a physical link $e_{i}$, it can be described as a node pair which is shown in Formula (2), where ${e_{i}}^{v_{a}}$ and ${e_{i}}^{v_{b}}$ represent the two nodes connected by link $e_{i}$.

(2) $$e_{i}=\left({e_{i}}^{v_{a}},{e_{i}}^{v_{b}}\right),\forall e_{i}\in E$$

Like the computing resources of physical nodes, the physical links also has certain bandwidth resources represented by $b\left(e_{i}\right)$, and $r\left(e_{i}\right)$ denotes the remaining bandwidth resources. We use $L\left(e_{i}\right)$ to represent the link load rate. The calculation of link load rate $L\left(e_{i}\right)$ is defined in Formula (3).

(3) $$L\left(e_{i}\right)=\frac{b\left(e_{i}\right)-r\left(e_{i}\right)}{b\left(e_{i}\right)},\forall e_{i}\in E$$

After the above definition, we use $p\left(v_{i},v_{j}\right)$ to denote a path between nodes $v_{i}$ and $v_{j}$. $p\left(v_{i},v_{j}\right)$ is a set which contains all physical links on this path. This can be defined in Formula (4).

(4) $$p\left(v_{i},v_{j}\right)=\left\{e_{m},\cdots ,e_{n}\right\}\subseteq E,\forall v_{i},v_{j}\in V$$

We use $D\left[p\left(v_{i},v_{j}\right)\right]$ to represent end-to-end delay of $p\left(v_{i},v_{j}\right)$. $D\left[p\left(v_{i},v_{j}\right)\right]$ consists of three parts: $d_{prop}$, $d_{proc}$, and $d_{trans}$, which can be represented in Formula (5). $d_{prop}$ means the propagation delay. Although we consider a multi-domain scenario, we assume that the distance between the domains is negligible, so we can regard $d_{prop}$ as 0. $d_{proc}$ means the packets process delay, which is not change so we can not consider it. So that the end-to-end delay equals to the transmission delay $d_{trans}$, which can also be denoted in Formula (6).

(5) $$D\left[p\left(v_{i},v_{j}\right)\right]=d_{prop}+d_{proc}+d_{trans}$$

(6) $$\begin{array}{c} D\left[p\left(v_{i},v_{j}\right)\right]=\sum {}_{e_{k}\in p\left(v_{i},v_{j}\right)}d\left(e_{k}\right),\forall v_{i},v_{j}\in V \end{array}$$

The above is for the modeling of the physical network, then we describe the modeling of SFC request.

### 4.2. SFC Request

In order to better describe SFC quantitatively, we can denote an SFC request as $SFC=\left(V_{S},E_{S},S,D,F\right)$. In which, $V_{S}=\left\{vnf_{1},vnf_{2},vnf_{3},\cdots ,vnf_{\left|VS\right|}\right\}$ represents the set of all the VNFs in SFC, similarly, $E_{S}=\left\{vl_{1},vl_{2},vl_{3},\cdots ,vl_{\left|ES\right|}\right\}$ represents the set of all the virtual links in SFC. $\left|VS\right|$ represent the number of VNFs, $\left|ES\right|$ represent the number of virtual links.

The computing resources a VNF request is denoted by $c\left(vnf_{i}\right)$. $V\left(vnf_{i}\right)$ represents the physical node where the VNF $vnf_{i}$ is deployed. We introduce a new variable $M\left(vnf_{i},v_{j}\right)$ which is a binary variable. If $vnf_{i}$ is successfully deployed on the node $v_{j}$, $M\left(vnf_{i},v_{j}\right)=1$; otherwise, $M\left(vnf_{i},v_{j}\right)=0$. We indicate it in Formula (7).

(7) $$M\left(vnf_{i},v_{j}\right)\in \left\{0,1\right\},\forall vnf_{i}\in V_{S},\forall v_{j}\in V$$

Similarly, we use $b\left(vl_{i}\right)$ to represent the bandwidth resources a virtual link $vl_{i}$ need to deploy on the physical link. We use $v{l_{i}}^{vnf_{a}}$ and $v{l_{i}}^{vnf_{b}}$ to represent the two VNFs connected by the virtual link $vl_{i}$. It can be represented by Formula (8)

(8) $$vl_{i}=\left(v{l_{i}}^{vnf_{a}},v{l_{i}}^{vnf_{b}}\right),\forall vl_{i}\in E_{S}$$

We use a binary variable $N\left(vl_{i},e_{j}\right)$ to describe the virtual link’s deployment situation. If virtual link $vl_{i}$ is successful deployed on the physical link $e_{j}$, $N\left(vl_{i},e_{j}\right)=1$; otherwise, $N\left(vl_{i},e_{j}\right)=0$. This can be summarized in Formula (9).

(9) $$N\left(vl_{i},e_{j}\right)\in \left\{0,1\right\},\forall vl_{i}\in E_{S},\forall e_{j}\in E$$

$p\left(vl_{i}\right)$ denotes the physical link where the virtual link $vl_{i}$ deployed on. $E\left(vl_{i}\right)$ represents the path which the virtual link $vl_{i}$ deployed on. $D\left(vl_{i}\right)$ represents the latency of $vl_{i}$, $B\left(vl_{i}\right)$ represents the bandwidth consumption of deploying the path of virtual link $vl_{i}$. Formulas (10)–(12) have shown the definition of the above variables, in which $NUM\left[E\left(vl_{i}\right)\right]$ represents the number of links on path $E\left(vl_{i}\right)$.

(10) $$E\left(vl_{i}\right)=p\left[V\left(v{l_{i}}^{vnf_{a}}\right),V\left(v{l_{i}}^{vnf_{b}}\right)\right],\forall vl_{i}\in E_{S}$$

(11) $$D\left(vl_{i}\right)=d\left[E\left(vl_{i}\right)\right]=\sum {}_{e_{k}\in E\left(vl_{i}\right)}d\left(e_{k}\right),\forall vl_{i}\in E_{S}$$

(12) $$\begin{array}{c} B\left(vl_{i}\right)=b\left[E\left(vl_{i}\right)\right]=b\left(vl_{i}\right)*NUM\left[E\left(vl_{i}\right)\right] \end{array}$$

### 4.3. Dynamic SFC Deployment

The source node *S* and destination node *D* of the SFC represent the location of the network service provider and the user, respectively. Besides, in the RFC7665 [30], an SFC service function chain defines an ordered set of abstract service functions and ordering constraints that must be applied to packets and/or frames and/or flows selected as a result of classification. Thus, we use *F* to describe the ordered flows in Formula (13).

(13) $$F=\left\{vnf_{1}\overset{vl_{1}}{⟶}vnf_{2}\overset{vl_{2}}{⟶}vnf_{3}\overset{vl_{3}}{⟶}\cdots \overset{vl_{\left|ES\right|}}{⟶}vnf_{\left|VS\right|}\right\}$$

During the deployment process of dynamic SFC, we will define some variables to measure the deployment situation and network performance. In the process of deployment, for an SFC $SFC_{i}$, we use $T_{a}\left(SFC_{i}\right)$ to represent the arrival time internal of the current SFC $SFC_{i}$ and the previous one $SFC_{i-1}$. $F_{s}\left(SFC_{i}\right)$ denotes the service time of $SFC_{i}$. In addition, $TR\left(SFC_{i}\right)$ represents the required time to respond to an SFC $SFC_{i}$.

In a complete SFC deployment process, we use $S_{SFC}$ to represent the set of all the SFC requests. For an SFC $SFC_{i}$, we use $DL\left(SFC_{i}\right)$ to represent end-to-end delay, $BW\left(SFC_{i}\right)$ to represent the bandwidth consumption of deploying an SFC $SFC_{i}$. The calculation of $DL\left(SFC_{i}\right)$ and $BW\left(SFC_{i}\right)$ are shown in Formulas (14) and (15).

(14) $$\begin{array}{c} DL\left(SFC_{i}\right)=\sum {}_{vl_{k}\in E_{S}}D\left(vl_{k}\right),\forall SFC_{i}\in S_{SFC} \end{array}$$

(15) $$BW\left(SFC_{i}\right)=\sum {}_{vl_{k}\in E_{S}}B\left(vl_{k}\right),\forall SFC_{i}\in S_{SFC}$$

We set a binary variable $P\left(SFC_{i}\right)$ to describe the SFC deployment situation. If the SFC $SFC_{i}$ is deployed successfully, $P\left(SFC_{i}\right)=1$, otherwise, $P\left(SFC_{i}\right)=0$. We can use Formula (16) to represent it.

(16) $$P\left(SFC_{i}\right)\in \left\{0,1\right\},\forall SFC_{i}\in S_{SFC}$$

In the whole process of dynamic SFC deployment process, all SFC requests can be represented by $S_{SFC}=\left\{SFC_{1},SFC_{2},SFC_{3},\cdots ,SFC_{\left|S_{SFC}\right|}\right\}$, $\left|S_{SFC}\right|$ is the number of SFC requests. $NUM_{suss}\left(S_{SFC}\right)$ is used to denote the number of SFCs successfully deployed. $DL_{all}\left(S_{SFC}\right)$ represents all the delay, whereas $BW_{all}\left(S_{SFC}\right)$ represents all the bandwidth consumption, respectively. $TR_{all}\left(S_{SFC}\right)$ denotes the response time of $S_{SFC}$. In the above definitions, we only consider SFCs that have been deployed successfully. These parameters can calculate by Formulas (17)–(20).

(17) $$\begin{array}{c} NUM_{suss}\left(S_{SFC}\right)=\sum {}_{SFC_{k}\in S_{SFC}}P\left(SFC_{k}\right) \end{array}$$

(18) $$DL_{all}\left(S_{SFC}\right)=\sum {}_{SFC_{k}\in S_{SFC}}DL\left(SFC_{k}\right)*P\left(SFC_{k}\right)$$

(19) $$BW_{all}\left(S_{SFC}\right)=\sum {}_{SFC_{k}\in S_{SFC}}BW\left(SFC_{k}\right)*P\left(SFC_{k}\right)$$

(20) $$TR_{all}\left(S_{SFC}\right)=\sum {}_{SFC_{k}\in S_{SFC}}TR\left(SFC_{k}\right)*P\left(SFC_{k}\right)$$

Because we mainly focus on the dynamic deployment of SFC, the service requests’ arrival and departure need to be taken into account. We assume that the dynamic arrival and departure of a service request is following the Poisson distribution. Therefore, the interval of arrival time and the length of service time are independently and identically distributed, and both of them obey an exponential distribution. The process of the above two parameters is shown in (21) and (22).

(21) $$\begin{array}{c} T_{a}\left(SFC_{i}\right)-T_{a}\left(SFC_{i+1}\right)=\frac{\log^{u}}{\lambda },u\in \left(0,1\right) \end{array}$$

(22) $$\begin{array}{c} F_{s}\left(SFC_{i}\right)-F_{s}\left(SFC_{i+1}\right)=\frac{\log^{v}}{\mu },v\in \left(0,1\right) \end{array}$$

In the whole process of SFC deployment, we use $LB\left(SFC_{i}\right)$ to represent the load balance rate of the physical network. It can be calculated by Formula (23), in which α (0<α<1) is a weighted factor.

(23) $$\begin{array}{c} LB\left(SFC_{i}\right)=\alpha *L\left(v_{i}\right)+\left(1-\alpha \right)*L\left(e_{i}\right) \end{array}$$

### 4.4. Network Resource Constraints

When deploying the VNF $vnf_{i}$ on a physical network node $V\left(vnf_{i}\right)$, we need to ensure that the computing resources required by $vnf_{i}$ should be less than the remaining computing resources $r\left[V\left(vnf_{i}\right)\right]$ on the physical node. This limitation is reflected in Formula (24). The consumed computing resources of node $v_{j}$ need to be less than the total computing resources of itself which is shown in Formula (25).

(24) $$r\left[V\left(vnf_{i}\right)\right]\geq c\left(vnf_{i}\right),\forall vnf_{i}\in V$$

(25) $$c\left(v_{j}\right)\geq \sum {}_{SFC_{k}\in S_{SFC}}\sum {}_{vnf_{i}\in V_{S}}M\left(vnf_{i},v_{j}\right)\times c\left(vnf_{i}\right),\forall v_{j}\in V$$

During the deployment process, each VNF in an SFC and physical node will be mapped one-to-one. This can simplify the SFC deployment scheme and reduce the problem of excessive load on one node, achieving better load balancing. This is shown is Formulas (26) and (27)

(26) $$\begin{array}{c} 0\leq \sum {}_{v_{j}\in V}M\left(vnf_{i},v_{j}\right)\leq 1,\forall vnf_{i}\in V_{S} \end{array}$$

(27) $$\begin{array}{c} 0\leq \sum {}_{vnf_{i}\in V_{S}}M\left(vnf_{i},v_{j}\right)\leq 1,\forall v_{j}\in V \end{array}$$

In the process of mapping virtual links, there are also some constrains need to be taken into account. For a virtual link $vl_{i}$ and the physical path $E\left(vl_{i}\right)$ it deployed on, we need to ensure that the bandwidth consumption of a virtual link $vl_{i}$ should be less than the remaining bandwidth resources $r\left[E\left(vl_{i}\right)\right]$ of the physical path $E\left(vl_{i}\right)$. This constrain is described in Formula (28). For the physical link $e_{j}$, the consumed bandwidth resources need to be less than the total bandwidth resources of itself. This can be shown in Formula (29). During the deployment of an SFC, one physical link $e_{j}$ only can deploy one virtual link $vl_{i}$, which can be shown in Formula (30).

(28) $$r\left[E\left(vl_{i}\right)\right]\geq b\left(vl_{i}\right),\forall vl_{i}\in E_{S}$$

(29) $$b\left(e_{j}\right)\geq \sum {}_{SFC_{k}\in S_{SFC}}\sum {}_{vl_{i}\in E_{S}}N\left(vl_{i},e_{j}\right)\times b\left(vl_{i}\right),\forall e_{j}\in E$$

(30) $$0\leq \sum {}_{vl_{i}\in E_{S}}N\left(vl_{i},e_{j}\right)\leq 1,\forall e_{j}\in E$$

## 5. Algorithm Design

In this section, we describe our proposed algorithm SFCDO for the dynamic SFC deployment. This algorithm has two purposes: (a) to find the shortest physical path for orchestration, (b) to get the best SFC deployment scheme based on the result of the previous step, so the algorithm we designed includes two algorithms: (a) the sequential traversal of the network topology based on Breadth-First Search, and (b) the determination of the deployment plan based on the Improved Ant Colony Optimization algorithm.

### 5.1. Breadth-First Search

The algorithm of the first step is the BFS algorithm, which is to find the shortest road between the source node and the destination node. The service terminal and the user are located on the nodes, respectively. BFS utilize the sequence traversal between the source node and the destination node to find the length of the shortest path between two nodes. In Algorithm 1, we describe the steps of this algorithm.

The input of Algorithm 1 is the physical network topology. We use an adjacency list to store the location information of nodes and links. Besides, we already know the start node and end nodes of an SFC request. This algorithm will output the length of the shortest path between the start node and the end node as a result.

Before starting, we need to perform initialization operations. We use *queue* for network traversal and path finding. The *queue* follows the first in first out principle, and we set it to empty at the beginning. In addition, we set some parameters for recording. The *queue* is the node currently arriving, *list1* records the node to be checked, *list2* records the node had been checked, *hop* records the path length when reaching the node, and *path* records the path topology information of the current node, *length* is the shortest path between node *S* and *D*.
**Algorithm 1** Breadth-First Search Algorithm**Require:** Physical network
G=(V,E); The source node *S*; The destination node *D*;
**Ensure:** The length of the shortest path between two nodes; The topology information of the shortest path.1:  **Initialization**: queue=⌀;2:  Push *S* into the queue;3:  **while**
queue≠⌀
**do**4:    Add the next node into list1;5:    Mark *S* as already checked;6:    Add *S* into list2;7:    hop=hop+1;8:     **if** *D* in the queue **then**9:     Put all nodes into list2;10:     Make list1 empty;11:     length=hop;12:     **return** length and path;13:    **end if**14:  **end while**

In line 2, we push the source node *S* into the *queue*. When the *queue* is not empty, then we start to traverse all the nodes of the next hop, at the same time add them to *list1* (line3–4). Next, in line 5–8, we can mark the nodes have been visited and put them into *list2*, let *hop* = *hop* + 1. We continue to traverse the unvisited nodes. In the following process, if the destination node *D* is visited, then the traversal is ended. All nodes are marked as visited and put into *list2*, making *list1* empty. Then they return the result, including the length of the shortest path between two nodes, and the topology information of the shortest path.

Through the Breadth-First Search algorithm, we can get the length and topology information of the shortest path we need in the physical network. This result will serve as an important basis for SFC deployment. We will deploy VNFs and virtual links based on the shortest physical path, including physical nodes and physical links. In addition, we use the output results to illustrate the strategy of SFC deployment in Algorithm 2.

As for the complexity of BFS algorithm, first we assume that the underlying physical network has m nodes and n links. Each node goes in and out a queue at most once, so that the total time of operating the queue is *O*($\left|M\right|$). The network topology is stored in the adjacent list, and BFS only scans the adjacency list of the node when it pops a queue. Each adjacency list is scanned at most once. Therefore, the total time for scanning the adjacent lists is *O*($\left|N\right|$). So the time complexity of Algorithm 1 is *O*($\left|M\right|+\left|N\right|$).

### 5.2. Improved Ant Colony Optimization

Ant Colony Optimization (ACO) is a metaheuristic algorithm. It is inspired by the collaborative behavior of a group of ants when they find the shortest path between their nest to the source of food [31]. The ant colony algorithm is a kind of swarm intelligence algorithm. It is a group of individuals that show intelligent behavior through mutual cooperation, thus providing a new possibility for solving complex problems. It is also a probabilistic algorithm used to find optimal paths. In our scenario, we can use the ACO algorithm to get the best SFC deployment scheme.

We will briefly explain the working principle of this algorithm as follows. Ants start from the specified source node *S* and release a specific concentration of pheromone every time they pass through a link. The shorter the path, the more ants will pass, so the pheromone concentration on these paths will be higher than others. After a specified number of iterations, we finally obtain an optimal path.

ACO relies on pheromone, which leads to the local optimal solutions. Therefore, we will use IACO (Improved Ant Colony Algorithm) algorithm, which is combined with genetic algorithm (GA). This algorithm is based on the cross mutation factor of GA, which improves the pheromone concentration setting and enhances the global search ability of traditional ACO.

The principle of the IACO is: in the early stage of the algorithm, we use GA to initialize the population. Because it has a fast initial convergence speed, it is easy to get a better solution. Next, the better solution will be input as the initial pheromone of the ACO, and then run the ACO algorithm. Because we use GA for preprocessing before, it is not easy to fall into the local optimum, and the optimal solution we get is closer to the actual optimal solution.

In IACO, the steps to generate the initial input of the ACO are as follows:

Step 1: Encoding. For a physical network with *n* nodes, we use an array of length *n* to represent an SFC deployment path of the network, and use random key encoding and decoding methods to encode it.
Step 2: Parameter initialization. Set the GA maximum iteration number to $G_{\max}$, the population size is the same as the number of physical nodes *m*, $p_{c}$ represents the crossover probability, $p_{m}$ represents the mutation probability, and set the appropriate value function, which is the reciprocal of the shortest distance between two nodes.
Step 3: Calculate the appropriate value and use the roulette rule to select. For *N* chromosomes in the current population, the probability of the *i*-th chromosome being selected is
  (31) $$P_{i}=\frac{\frac{1}{f\left(x_{i}\right)}}{\sum_{j}^{N}\frac{1}{f\left(x_{j}\right)}}$$
  Among them, $f\left(\cdot \right)$ represents the objective function, which is the path length corresponding to the solution $x{}_{i}$.
Step 4: Adopt linear crossover. For two chromosomes $x{}_{i}$ and $x{}_{j}\left(i=j\right)$, linear crossover is implemented as follows:
  (32) $$x{}_{id}=\lambda \cdot x{}_{id}+\left(1-\lambda \right)\cdot x{}_{jd},i=1,2,\cdots ,N;d=1,2,\cdots ,D$$
  Among them, λ represents a random number in the interval of [0,1].
Step 5: Gaussian mutation of the GA algorithm. For a chromosome $x{}_{i}$, its variant is
  (33) $$x^{\prime }{}_{id}\leftarrow G\left(x_{id},\frac{1}{n}\right),d=1,2,\cdots ,D$$
Step 6: Start iteration, if the maximum iteration number is reached, then run the ACO section, otherwise, skip to Step 3.

After getting the optimized solution by the above steps, we can execute the ACO algorithm. The algorithm procedure is as follows:
**Algorithm 2** Ant Colony Optimization Algorithm**Require:** Physical network G=(V,E); The source node *S*; The destination node *D*;**Ensure:** The best deployment path Bestscheme;1:  **Initialization**: t=0, Bestlength→∞, Besttour=0, Delta=0, Tabu=0, Allowed=0;2:  **for** each link **do**3:    set initial pheromone p from GA algorithm;4:  **end for**5:  Add all the nodes into Allowed;6:  **for** each ant k **do**7:    randomly choose an initial city;8:    Add source node *S* into Tabu;9:    Delete *S* in the Allowed;10:    **for** i = 1 to n **do**11:     Search for the next node *T* by Formula (34);12:     Add *T* into Tabu;13:     Delete *T* in the Allowed;14:    **end for**15:  **end for**16:  Add source node *S* into Tabu;17:  Calculate the heuristic factor Delta by Formula (35);18:  Calculate the Tourlength;19:  **if**
Tourlength<Bestlength
**then**20:   Bestlength=Tourlength;21:   Besttour=Tabu; 22:  **else**23:   continue;24:  **end if**25:  NUM = NUM+1;26:  t=t+nt;27:  Update the matrix *P* by Formula (36);28:  **if** NUM = MAX-GEN **then**29:   **return**
Bestscheme;30:  **else**31:   Reinitialization;32:  **end if**

Just like Algorithm 1, we need to perform initialization operations before starting to execute it. We have specified some parameter variables as follows. *m* denotes the number of ants, the pheromone between all nodes is represented by the matrix *P*, *Bestlength* denotes the shortest path, *Bestscheme* denotes the best deployment path. *Tabu* is a table to store all the nodes have been visited, *Allowed* is another table to store the nodes unvisited. Matrix *Delta* stores the pheromone in a loop. The cost of the whole path is represented by *Tourlength*. We assume that the algorithm runs times is NUM, the total runs times is MAX-GEN, and the running time is *t*.

As shown in Algorithm 2, this algorithm starts at the source node *S* in the physical network. Line1–4 represents the initialization of all parameters and the processing of the source node *S*. Then we search the next node and calculate the heuristic factors. The probability of the next node we choose is calculated by Formula (34). The heuristic factor is calculated by Formula (35).

(34) $$p_{ij}^{k}\left(t\right)=\left\{\begin{array}{cc} \frac{{\left[\tau_{ij}\left(t\right)\right]}^{\alpha }\cdot {\left[\eta_{ij}\left(t\right)\right]}^{\beta }}{\sum_{s\in J_{k}\left(i\right)}{\left[\tau_{ij}\left(t\right)\right]}^{\alpha }\cdot {\left[\eta_{ij}\left(t\right)\right]}^{\beta }} & \mathrm{if}\;j\in J_{k}\left(i\right);\\ 0 & \mathrm{otherwise}. \end{array}\right.$$

(35) $$\eta_{ij}=\frac{1}{d_{ij}}$$

In the Formula (34), $\tau_{ij}\left(t\right)$ represents the pheromone concentration of node i and j at time t, whereas $\eta_{ij}\left(t\right)$ represents the visibility from node i and j. α and β represent the weight of pheromone concentration and heuristic factor, respectively. In the Formula (35), $d_{ij}$ is the distance from node i to node j. Therefore, the smaller the $d_{ij}$, the larger the $\eta_{ij}$.

The cost of the whole path used to compare with the *Bestlength* in order to get the result of the shortest path. Next, we will continue to iterate, at the same time, we update the matrix *P* by Formula (36). $\tau_{ij}\left(t+n\right)$ represents the pheromone concentration between node i and j at time t+n. The calculation of parameters $\Delta \tau_{ij}$ and $\Delta {\tau_{ij}}^{k}$ are shown in Formulas (37) and (38). $\Delta {\tau_{ij}}^{k}$ represents the pheromone left by ant k between node i and j. $L_{k}$ represents the total distance of the path traveled by ant k through a iteration, namely *Tourlength*.

(36) $$\tau_{ij}\left(t+n\right)=\left(1-\rho \right)\cdot \tau_{ij}\left(t\right)+\Delta \tau_{ij}$$

(37) $$\Delta \tau_{ij}=\sum_{k=1}^{m}\Delta {\tau_{ij}}^{k}$$

(38) $$\Delta \tau_{ij}^{k}=\left\{\begin{array}{cc} \frac{Q}{L_{k}} & \mathrm{if}\;\mathrm{ant}\;k\;\mathrm{passes}\;\mathrm{link}\;\left(i,j\right)\;\mathrm{in}\;\mathrm{this}\;\mathrm{tour};\\ 0 & \mathrm{otherwise}. \end{array}\right.$$

Next, we repeat the above algorithm steps until the number of iterations reaches the maximum number of runs and output the best deployment path *Besttour*.

Note that while the above formulation and algorithms focus on initial deployment, they can also be used for dynamic adjustment of deployment. In particular, the SR controller in the system regularly collects network-related information, such as link delay, bandwidth resource utilization, and so on, in order to re-run the above procedures to obtain the latest and best deployment scheme.

### 5.3. Algorithm Performance Discussion

Next, we will discuss the scalability, running time and computational complexity of our algorithm. In terms of scalability, our algorithm has been verified in the simulation part that it reduces end-to-end delay in both small topology and large topology networks, and has good scalability. At the same time, if a large number of SFC requests arrive, the IACO heuristic algorithm can also dynamically adjust the deployment plan according to the request, ensuring the effectiveness of the algorithm.

In terms of running time, in order to simplify the analysis and eliminate the influence of the external environment, the time complexity of the algorithm is generally discussed to demonstrate the pros and cons of the running time. In the two-step algorithm in this paper, the time complexity of BFS algorithm is $O\left(m^{2}\right)$, the time complexity of IACO is Om·m−1·n·TT22. The highest order item is $n{}^{2}$, so the time complexity is within the acceptable range.

Another measure of computational complexity is space complexity. The space complexity of BFS algorithm is $O\left(m+l\right)$, the space complexity of IACO is $O\left(m^{2}+m\cdot n\right)$, in which m is the number of physical nodes, l is the number of physical links, n is the number of ants, T is iteration numbers.

## 6. Performance Evaluation

In this section, we perform many simulation experiments to evaluate the capability of our proposed algorithm and compare its performance with the other three existing algorithms: Greedy-Simulated Annealing (G-SA) algorithm [16], SFC deployment optimization algorithm [32], and Bandwidth Optimization Algorithm ER_CS_ADJ [18]. The G-SA and ER_CS_ADJ algorithm are introduced in Section 2. The SFC deployment optimization algorithm adpoted BFS to search for the shortest path and deploy VNFs based on physical resources constraints, so we regard it as the SFC-BFS algorithm.

The three comparison algorithms we chose are: G-SA, SFC-BFS, and ER_CS_ADJ. The reason for choosing G-SA is that it and SFCDO are both two-step algorithms, and they have part of the heuristic algorithm, which enables a better contrast. SFC-BFS is chosen because it and SFCDO also have BFS but the SFC generation methods are different, which can get the optimized result for IACO. ER_CS_ADJ considered the bandwidth resource consumption and load balancing, which is similar to SFCDO. Thus, we chose these three algorithms to undertake a complete comparison with the proposed algorithm according to different aspects.

### Simulation Settings

In order to evaluate the performance of the deployment algorithm described in Section 6, we use Java to implement a simulation environment. To prove the performance of the proposed algorithm in the SFC deployment problem, similar to Ref. [18], we adopt the Waxman 2 model from the GT-ITM [33] to randomly generate small and large network instances as physical networks for SFC deployment. In this paper, we assume that the small physical network contains 50 nodes and the large physical network includes 200 nodes, respectively. We chose a machine with an Intel i7 CPU with 9.8 GB of RAM.

Similar to Ref. [34], we assume that each node contains 1500 units of computing resources, and we set each link to include 1500 units of bandwidth resources. For a physical link $e_{i}$, the delay $d\left(e_{i}\right)$ obeys a uniform distribution U(10,20). For both small and large network topologies, we set the arrival rate of service request λ to 0.04. For the small topology, the service rate μ is set to $2\times 10^{-4}$, whereas for the large topology, the service rate μ is set to $5\times 10^{-5}$. For a VNF $vnf_{i}$, the computing resources requested by it $c\left(vnf_{i}\right)$ follows the same uniform distribution U(10,20). For a virtual link $vl_{i}$, the bandwidth resources to be requested follows the uniform distribution U(20,40).

In this paper, the optimization goals we choose are as follows: (*i*) average end-to-end delay, (*ii*) average bandwidth resources consumption, (*iii*) maximum node load rate, (*iv*) maximum link load rate. Calculation of these parameters are shown in Formulas (39)–(42).

(*i*) average end-to-end delay

(39) $$D_{average}=\frac{DL_{all}\left(S_{SFC}\right)}{NUM_{suss}\left(S_{SFC}\right)}$$

(*ii*) average bandwidth resources consumption

(40) $$B_{average}=\frac{BW_{all}\left(S_{SFC}\right)}{NUM_{suss}\left(S_{SFC}\right)}$$

(*iii*) maximum node load rate

(41) $$VR_{\max}=\max_{v_{k}\in V}\left\{L\left(v_{k}\right)\right\}$$

(*iv*) maximum link load rate

(42) $$ER_{\max}=\max_{e_{k}\in E}\left\{L\left(e_{k}\right)\right\}$$

The reasons why we chose the above four performance comparisons are as follows. Deploying SFC is to provide users with end-to-end services efficiently since, especially for multi-domain SFC orchestration, end-to-end delay must be considered. SFC deployment is not only the mapping of virtual nodes to physical nodes, but also the mapping of virtual links to physical links, so the bandwidth resource needs to be considered. The success of SFC deployment is connected with the network load balancing, so we consider the load rate of the nodes and links to obtain an effective conclusion of the optimization effect.

**Figure 5:**
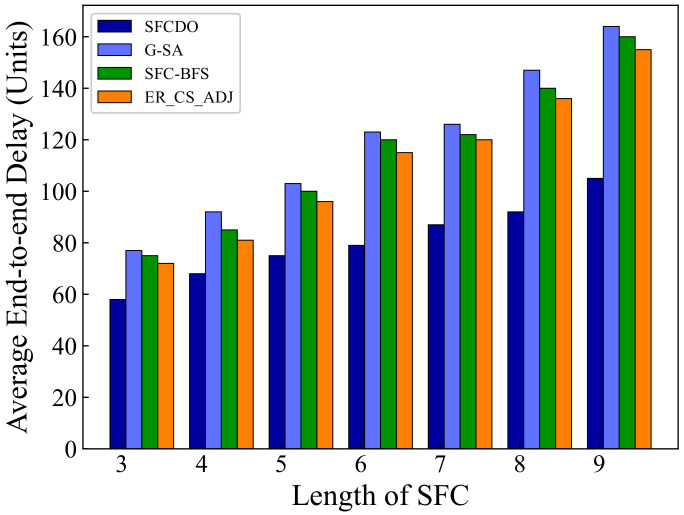
Average end-to-end delay of SFCs in small topology.

**Figure 6:**
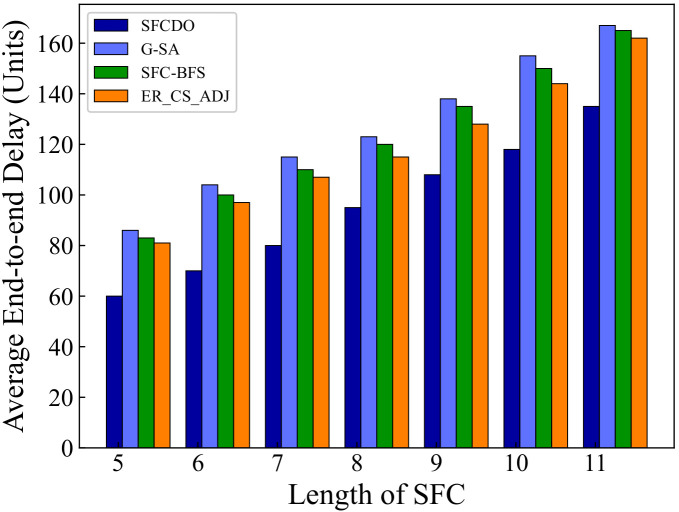
Average end-to-end delay of SFCs in large topology.

As shown in Figure 5 and Figure 6, with the increase of the SFC length, the end-to-end delay also increases. In general, for the proposed algorithm in this paper and the other three algorithms, the growth rate of latency is almost the same as that of the underlying physical links. The end-to-end delay of SFCDO is always less than that of comparison algorithms, both in small topology and large topology. The reason for this is that in SFCDO, we often choose the shorter path with fewer hops to deploy SFC, so that we can efficiently reduce the end-to-end delay than other algorithms. Our algorithm optimizes this factor by 24% in small topology and 20% in large topology.

**Figure 7:**
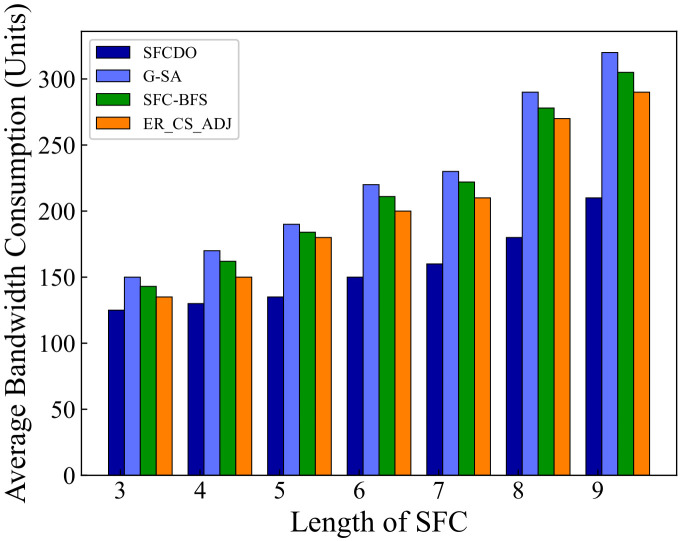
Average bandwidth consumption of SFCs in small topology.

**Figure 8:**
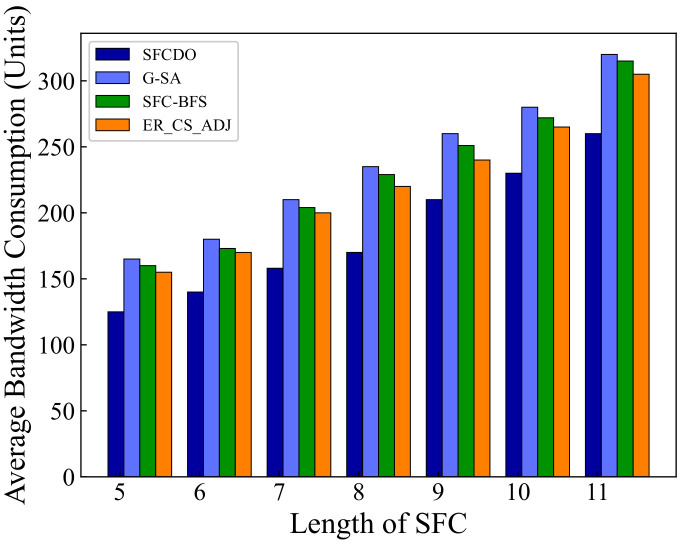
Average bandwidth consumption of SFCs in large topology.

In Figure 7 and Figure 8, we compare the average bandwidth resources consumption of SFCDO and other algorithms. In both small topology and large topology, with the length of SFC increasing, the bandwidth consumption increases. In the four algorithms, the average increase of this parameter is approximately equal to the average bandwidth resource required by a virtual link. However, It is not difficult to find that the bandwidth consumption of SFCDO is always smaller than the other three algorithms. This is because, when we select the physical links to deploy virtual links, the BFS algorithm we used will help choose the path with the minor links so that the average bandwidth resource consumption of deploying SFC will be reduced accordingly. SFC-BFS algorithm also uses BFS to obtain the shortest path, but it deploys VNFs directly according to the bandwidth resources constraints, which is less flexible than SFCDO, and therefore consumes bandwidth slightly higher than SFCDO. Our algorithm optimizes this indicator by 21% in the small topology and 15% in the large topology.

Next, we will introduce the performance of the four algorithms in terms of load rate. The parameters include the maximum node load rate and the maximum link load rate.

**Figure 9:**
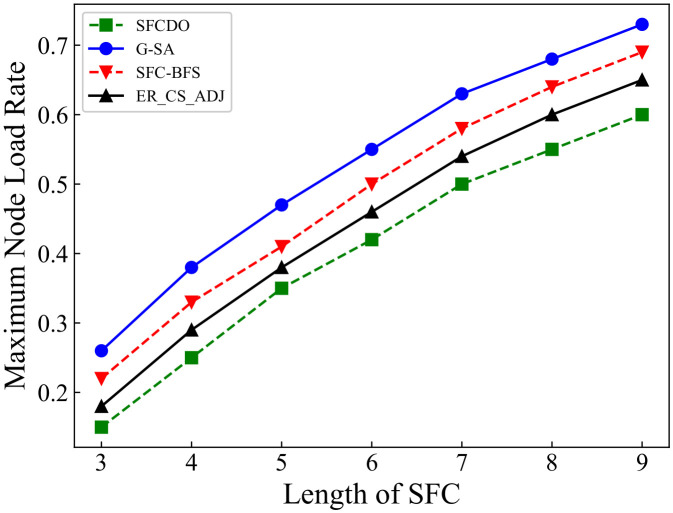
Maximum node load rate of SFCs in small topology.

**Figure 10:**
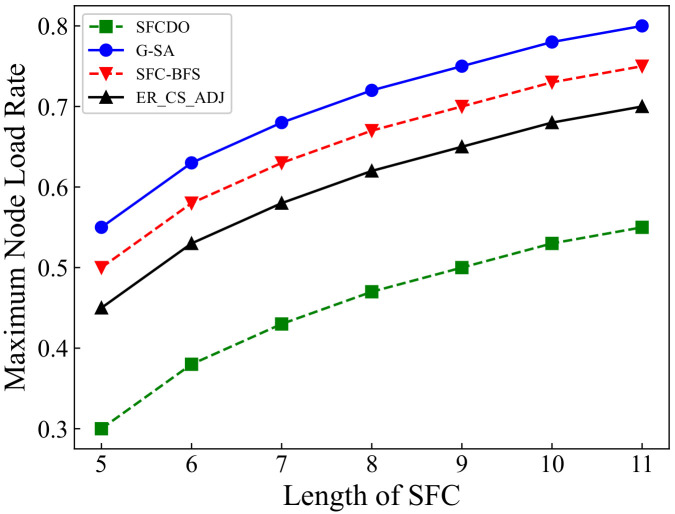
Maximum node load rate of SFCs in large topology.

As shown in Figure 9 and Figure 10, the growth rate of SFCs maximum node load rate in two different topologies is similar. In both small topology and large topology, the maximum node load rate increases with the increase of the SFC length. When the SFC length remains the same, the value of the maximum node load rate in SFCDO is always lower than that of the three other algorithms. The reason for this is that neither SFC-BFS nor ER_CS_ADJ dynamically adjust the next VNF deployment step based on the current node load. G-SA can dynamically deploy SFC deployment based on the simulated annealing process, but its performance is still worse than the SFCDO because its global search ability for the optimal solution is not as good as the improved ACO algorithm.

**Figure 11:**
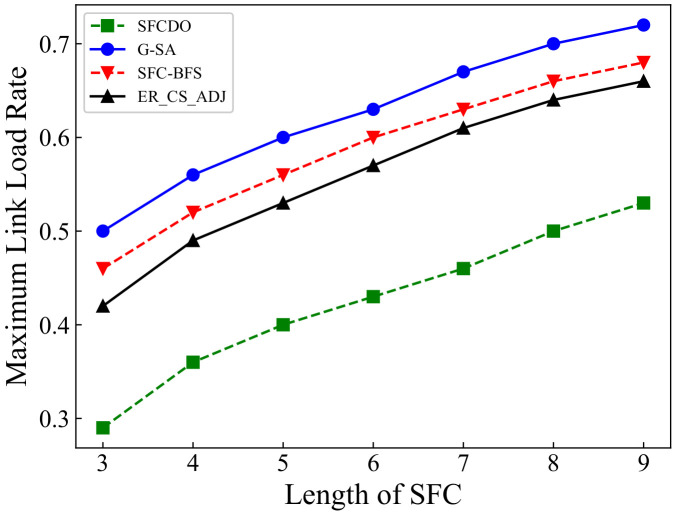
Maximum link load rate of SFCs in small topology.

**Figure 12:**
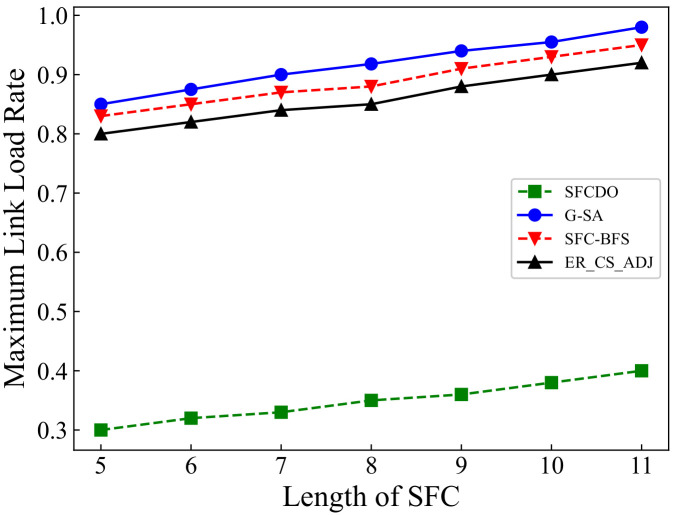
Maximum link load rate of SFCs in large topology.

In Figure 11 and Figure 12 we show the results of maximum link load rate in two different topologies. With the length of SFC increases, the maximum link load rate also increases. In general, the other three algorithms’ curves are always higher than those of SFCDO, which is more evident in large-scale topology. This is because, in our algorithm, when we deploy each VNF, we will consider preferential deployment on nodes with relatively low load rate by IACO, so the links that pass through also have lower load rate. For other algorithms, because the VNF will be deployed at the central node, the node and link load rates are always higher than SFCDO.

It is mentioned in Ref. [35] that mapping is similar to embedding, so this article considers mapping performance. Migration is a kind of mapping. This article uses virtual network migration to save energy. Although it considers the reconfiguration cost incurred during virtual routing migration, it is also a choice for a constrained mapping process, which can be used for reference and partly applied to the scenarios mentioned in this article. The second step of the algorithm proposed in this paper is a heuristic algorithm, which can take the conditional constraints and state transition conditions of virtual node migration as the initial state of the algorithm, and obtain the optimal mapping scheme for virtual node migration after multiple iterations. According to the new optimal mapping scheme, the VNF can be re-deployed on the physical node, the virtual link is mapped to the physical link, and the reconfiguration cost can be recalculated. The original text considers that traffic is directed through VNFs in order to form SFCs to provide services. Ref. [35] considers minimizing the cost of virtual node migration to release more physical resources. Therefore, the original text can set physical resources to be migrated to increase the flexibility of large SFC deployment and the utilization of the physical network.

At the same time, there are many aspects that can be discussed about dynamic SFC deployment, such as the allocation of bandwidth resources in the physical network after a large number of SFCs are deployed, and the security of information in multi-domain orchestration. The authors of [36] demonstrated the performance of resource allocation for The Nash Bargaining Solution. This work has made a useful exploration of the solution to the resource allocation problem. The authors of [37] proposed a platform called telecommunication network as a service (TaaS), analyzing an open platform for network functions virtualization (OPNFV) activities and compared them with the TaaS concept to find commonalities and see how well it addresses security concerns outlined for TaaS. This article mainly discusses the security issues of virtualized networks. Referring to the above two related works, we will consider discussing these two problems in our future work.

## 7. Conclusions and Future Work

In this paper, we considered the dynamic orchestration and deployment of SFC combined with the SRv6 mechanism. First, we described ETSI NFV architecture for SFC orchestration. Then we illustrated the working process of the SFC orchestration and deployment leveraging SRv6 in a multi-domain scenario. Next, we proposed a two-step algorithm to improve the performance of the deployment process, including end-to-end delay, bandwidth consumption, and its load balancing. The first step is to choose the shortest path discovery algorithm Breadth-First Search to get the shortest path on the physical network to prepare for the subsequent SFC deployment. In the second step, we adopted the IACO algorithm to get the deployment scheme and iterated to get the best results. Finally, we compared our proposed algorithm with three previous algorithms. Experimental results demonstrated that the algorithm we proposed can reduce the end-to-end delay, bandwidth consumption and improve the load balancing effect.

In the future study of SFC deployment problems, owing to the emergence and application of new network communication architectures such as 5G, 6G, and intent-based networks, advanced technologies such as machine learning and deep learning will be integrated into SFC deployment problems to analyze users’ needs and translate users’ intent. In this paper, we do not take into account the intent translation of user service requests. In the future, we will try to utilize deep reinforcement learning technology to achieve the transformation of users’ intent and combined it with SFC orchestration and deployment to optimize its overall performance under new network architectures.

## Data Availability

Not applicable.

## References

[1] Yi B., Wang X., Li K., Huang M. (2018). A comprehensive survey of network function virtualization. Comput. Netw..

[2] Do T.-X., Kim Y. Latency-Aware Placement for State Management Functions in Service-Based 5G Mobile Core Network. Proceedings of the 2018 IEEE Seventh International Conference on Communications and Electronics (ICCE).

[3] Toumi N., Bernier O., Meddour D.E., Ksentini A. (2021). On cross-domain Service Function Chain orchestration: An architectural framework. Comput. Netw..

[4] Sun G., Li Y., Liao D., Chang V. (2018). Service Function Chain Orchestration across Multiple Domains: A Full Mesh Aggregation Approach. IEEE Trans. Netw. Serv. Manag..

[5] Huin N., Tomassilli A., Giroire F., Jaumard B. (2018). Energy-Efficient Service Function Chain Provisioning. J. Opt. Commun. Netw..

[6] Savi M., Tornatore M., Verticale G. Impact of processing costs on service chain placement in network functions virtualization. Proceedings of the IEEE Conference on Network Function Virtualization and Software Defined Network (NFV-SDN).

[7] Gupta A., Habib M., Chowdhury P., Tornatore M., Mukherjee B. On service chaining using virtual network functions in network-enabled cloud systems. Proceedings of the IEEE International Conference on Advanced Networks and Telecommuncations Systems (ANTS).

[8] Gupta A., Mukherjee B., Jaumard B., Tornatore M. Service chain (SC) mapping with multiple SC instances in a wide area network. Proceedings of the IEEE Global Telecommunications Conference-GLO BECOM.

[9] Paolucci F. (2018). Network Service Chaining Using Segment Routing in Multi-Layer Networks. J. Opt. Commun. Netw..

[10] Troia S., Cibari A., Alvizu R., Maier G. (2020). Dynamic programming of network slices in software-defined metro-core optical networks. Opt. Switch. Netw..

[11] Dab B., Fajjari I., Rohon M., Auboin C., Diquelou A. Cloud-Native Service Function Chaining for 5G Based on Network Service Mesh. Proceedings of the ICC 2020–2020 IEEE International Conference on Communications (ICC).

[12] Sun G., Li Y., Li Y., Liao D., Chang V. (2018). Low-latency orches tration for workflow-oriented service function chain in edge computing. Future Gener. Comput. Syst..

[13] Troia S., Alvizu R., Maier G. (2019). Reinforcement Learning for Service Function Chain Reconfiguration in NFV-SDN Metro-Core Optical Networks. IEEE Access..

[14] Zhang X., Li Q., Zhang Z., Wu J., Yang J. (2021). vSFC: Generic and Agile Verification of Service Function Chains in the Cloud. IEEE/ACM Trans. Netw..

[15] Kuo T., Liou B.-H., Lin K.C.-J., Tsai M.-J. (2018). Deploying chains of virtual network functions: On the relation between link and server usage. IEEE/ACM Trans. Netw..

[16] Liu J., Li Y., Zhang Y., Su L., Jin D. (2017). Improve service chaining performance with optimized middlebox placement. IEEE Trans. Serv. Comput..

[17] Hu Y., Li T. Enabling efficient network service function chain deployment on heterogeneous server platform. Proceedings of the IEEE International Symposium on High Performance Computer Architecture (HPCA).

[18] Sun J., Zhu G., Sun G., Liao D., Li Y., Sangaiah A.K., Ramachran M., Chang V. (2018). A reliability-aware approach for resource efficient virtual network function deployment. IEEE Access..

[19] Boyuan Y., Yongli Z., Xiaosong Y. (2020). Service Function Path Provisioning with Topology Aggregation in Multi-Domain Optical Networks. IEEE/ACM Trans. Netw..

[20] Joshi K.D., Kataoka K. (2020). Psmart: A Lightweight, Privacy-Aware Service Function Chain Orchestration In Multi-Domain Nfv/Sdn. Comput. Netw..

[21] Kaur K., Mangat V., Kumar K. (2020). A Comprehensive Survey of Service Function Chain Provisioning Approaches in SDN and NFV Architecture. Comput. Sci. Rev..

[22] Medhat A.M., Carella G.A., Pauls M., Monachesi M., Corici M., Magedanz T. Resilient orchestration of service functions chains in a NFV environment. Proceedings of the 2016 IEEE Conference on Network Function Virtualization and Software Defined Networks, NFV-SDN.

[23] Shin S.M., Kwon G.I. (2017). Real-time monitoring technique using SFC classifier in NFV environment. Int. J. Appl. Eng. Res..

[24] Filsfils C., Previdi S., Ginsberg L., Decraene B., Litkowski S., Shakir R. (2018). Segment Routing Architecture. Internet Requests for Comments.

[25] 3GPP TS 23.501 V15.5.0. System Architecture for the 5G System, Release 15. 3GPP, March 2019. https://www.etsi.org/deliver/etsi_ts/123500_123599/123501/15.05.00_60/ts_123501v150500p.pdf.

[26] Wang D., Sun T. (2018). Service-Based Architecture in 5G.

[27] Ventre P.L., Tajiki M.M., Salsano S., Filsfils C. (2018). SDN Architecture and Southbound APIs for IPv6 Segment Routing Enabled Wide Area Networks. IEEE Trans. Netw. Serv. Manag..

[28] Mayer A., Salsano S., Ventre P.L., Abdelsalam A., Chiaraviglio L., Filsfils C. An Efficient Linux Kernel Implementation of Service Function Chaining for Legacy VNFs Based on IPv6 Segment Routing. Proceedings of the 2019 IEEE Conference on Network Softwarization (NetSoft).

[29] Clad F., Xu X., Filsfils A.C., Bernier C.D. (2018). Segment Routing for Service Chaining: Draft-Xu-Clad-Spring-Sr-Service-Chaining-00.

[30] Halpern J., Pignataro C. (2015). Service Function Chaining (SFC) Architecture. Internet Requests for Comments.

[31] Deneubourg J.L., Pasteels J.M., Erhaeghe J.C.V. (1983). Probabilistic behaviour in ants: A strategy of errors?. J. Theor. Biol..

[32] Sun G., Xu Z., Yu H., Chen X., Chang V., Vasilakos A.V. (2020). Low-Latency and Resource-Efficient Service Function Chaining Orchestration in Network Function Virtualization. IEEE Internet Things J..

[33] Calvert K.L., Zegura E. Gt-itm: Georgia Tech Internetwork Topology Models (Software). Georgia Tech. http://www.cc.gatech.edu/fac/Ellen.Zegura/gt-itm/gt-itm.tar.gz.

[34] Zhao D., Ren J., Lin R., Xu S., Chang V. (2019). On Orchestrating Service Function Chains in 5G Mobile Network. IEEE Access.

[35] Eramo V., Miucci E., Ammar M. (2014). Study of Migration Policies in Energy-Aware Virtual Router Networks. IEEE Commun. Lett..

[36] Nikolaevskiy I., Lukyanenko A., Gurtov A. (2017). Nash Bargaining Solution Allocation is Not Suitable for Datacenter Jobs. Int. Game Theory Rev..

[37] Monshizadeh M., Khatri V., Gurtov A. NFV Security Considerations for Cloud-Based Mobile Virtual Network Operators. Proceedings of the 24th International Conference on Software, Telecommunications and Computer Networks (SoftCOM).

